# STS-BN: An efficient Bayesian network method for detecting causal SNPs

**DOI:** 10.3389/fgene.2022.942464

**Published:** 2022-09-15

**Authors:** Yanran Ma, Botao Fa, Xin Yuan, Yue Zhang, Zhangsheng Yu

**Affiliations:** ^1^ Department of Bioinformatics and Biostatistics, School of Life Sciences and Biotechnology, Shanghai Jiao Tong University, Shanghai, China; ^2^ Department of Biochemistry and Molecular Biology, School of Basic Medical Sciences, Xi’an Jiaotong University, Xi’an, China

**Keywords:** Bayesian network, GWAS, two-stage method, epistasis, complex disease

## Abstract

**Background:** The identification of the causal SNPs of complex diseases in large-scale genome-wide association analysis is beneficial to the studies of pathogenesis, prevention, diagnosis and treatment of these diseases. However, existing applicable methods for large-scale data suffer from low accuracy. Developing powerful and accurate methods for detecting SNPs associated with complex diseases is highly desired.

**Results:** We propose a score-based two-stage Bayesian network method to identify causal SNPs of complex diseases for case-control designs. This method combines the ideas of constraint-based methods and score-and-search methods to learn the structure of the disease-centered local Bayesian network. Simulation experiments are conducted to compare this new algorithm with several common methods that can achieve the same function. The results show that our method improves the accuracy and stability compared to several common methods. Our method based on Bayesian network theory results in lower false-positive rates when all correct loci are detected. Besides, real-world data application suggests that our algorithm has good performance when handling genome-wide association data.

**Conclusion:** The proposed method is designed to identify the SNPs related to complex diseases, and is more accurate than other methods which can also be adapted to large-scale genome-wide analysis studies data.

## 1 Introduction

Recently, the development of high throughput technology provides the possibility of genome-wide association studies (GWAS), and the investigation of associated single nucleotide polymorphisms (SNPs) is common. The original idea of GWAS is to genotype individuals from the case group and control group, respectively, compare the distributions of SNPs between these two groups and identify the SNPs associated with the disease (Barrett et al., 2014). But this method can only estimate a single locus at a time, which is not suitable for complex diseases. Different from the simple Mendelian disorder, there are always mass factors that influence complex diseases such as gene-gene interactions and gene-environment interactions. In many cases, the effects of genes are multi-locus and indeterminate. In such situations, single-locus analysis methods are likely to leave out some epistatic interactions. However, in the face of the epistatic interaction problem, dealing with all the possible combinations of SNPs can be an extremely time-consuming task. Therefore, designing robust and effective methods for multi-locus analysis is highly desired. Under the circumstances, we proposed a score-based two-stage Bayesian network (STS-BN) method for detecting causal SNPs, which is more accurate and stable compared to other existing methods.

Traditional methods for multi-locus analysis include a series of statistical approaches. A commonly used method is logistic regression, which has the advantage of model interpretability. But considering the model complexity, it's impractical to deal with the high-dimensional covariates and interactions. Moreover, with the exponential increase of possible combinations, more samples are needed to ensure the stability of the estimation of interaction effects (McKinney et al., 2006). In this situation, Park and Hastie proposed a new logistic regression method with a penalization on the size of the L2-norm of the coefficients to improve the adaptability of logistic regression methods for the SNP data (Park and Hastie, 2008). However, the time-consuming parameter estimation process is still a significant limitation (Han et al., 2010). The predictor-based design also makes this method easy to include false positives (Han and Chen, 2011). To speed up the computational process, BOOST combined the likelihood ratio test with a Boolean operation-based and multi-stage design (Wan et al., 2010). But this method can only detect the interaction between two loci, which limits its practical value. Multifactor dimensionality reduction (MDR) is also a popular method which constructs a contingency table for every possible SNPs combination (Ritchie et al., 2003). However, MDR-based methods with single-objective function might not yield favorable results due to potential model preferences and disease complexities. Therefore, a multiobjective MDR (MOMDR) method was proposed to improve detection success rates (Yang et al., 2018). Apart from these, some Bayesian methods were also developed. Early in 2004, Wacholder et al. mentioned that when analyzing a SNP, Bayesian methods could help us reduce the false positives due to the strategy of declaring statistical significance based on a *p*-value alone (Wacholder et al., 2004). Furthermore, Bayesian approaches can incorporate prior knowledge and quantify all information and uncertainties in the form of posterior distributions. Then considering the epistasis interaction, Zhang and Liu proposed the BEAM (Bayesian epistasis association mapping) algorithm (Zhang and Liu, 2007). This algorithm contains a Bayesian epistasis inference tool implemented via Markov chain Monte Carlo (MCMC) and the B statistic for evaluating statistical significance. The combination of two statistical tools from different schools of statistics gives users a comprehensive and complementary perspective. In addition, Bayesian methods have been further extended to more complex analyses, such as GWAS meta-analysis (Sun et al., 2022).

An alternative idea is to use machine learning methods. Chen et al. proposed four support vector machine (SVM) based algorithms to solve feature selection problems when detecting gene-gene interactions: SVM-RFA, SVM-RFE, SVM-Local, and SVM-GA (Chen et al., 2008). The first two approaches adopt the greedy search strategy. They build a set of nested feature subsets by adding or removing one gene at a time, based on the prediction accuracy at each iteration. SVM-local keeps searching the neighborhood of the current solution set to choose the best feature set. But this usually only reaches a local optimum (Chen et al., 2008). SVM-GA introduces genetic algorithms to complete the search process. In most cases, the determination of the optimal solution of machine learning methods is based on the prediction accuracy. But this cannot guarantee that the association is true (Han et al., 2010). Sometimes the addition of more loci is inclined to improve the accuracy of prediction, but leads to a higher false positive rate. Therefore, some approaches will perform statistical tests after the machine learning sections. For example, in the method proposed by Jiang et al. (2009), the B statistic is adopted to declare the statistical significance that the candidate SNPs are associated with the disease.

Utilizing network structure to estimate the relationship between variables is also a feasible idea (Han et al., 2010; Han and Chen, 2011; Yilmaz et al., 2019). Especially, Bayesian network is a common-used tool with a relatively strict theoretical basis. It is proposed by Pearl (1985). Then in the late 1980s, Pearl (1988) and Neapolitan (1990) summarized the relevant properties of Bayesian network and made it a new research field. In recent years, the application of Bayesian network has become more popular with many successful examples, such as analyzing gene expression data, predicting protein-protein interactions, and so on (Su et al., 2013; Lyu et al., 2021). Currently, several Bayesian network methods have been developed to detect epistatic interactions from GWAS data (Han et al., 2010; Han and Chen, 2011; Peng et al., 2021). For example, Han et al. adopted this concept into their algorithm, DASSO-MB, to investigate the Markov blanket of the disease in the Bayesian network and infer the associated loci (Han et al., 2010). Numerical experiments have shown that their method can reduce the rate of false positives. A similar idea was also used in the MBRFS algorithm (Li et al., 2016). Their research indicates that $G^{2}$ statistic used in DASSO-MB stratifies the conditioned SNPs already selected in Markov blanket, which means that the addition of SNPs into the Markov blanket leads to the exponential growth of the number of stratifications. Hence, they adopt a repeated-fishing strategy to make sure the $G^{2}$ statistic can always hold a relative high power. But the structure learning method used in DASSO-MB relies on the independence test and makes the algorithm sample-consuming. Thus, when the sample size is insufficient, the stability of the algorithm may be affected to some extent. Later, a score-based Bayesian network approach, bNEAT, was designed to deal with the small sample data (Han and Chen, 2011). This method has shown its excellent performance when managing the small sample data. What’s more, it has higher accuracy compared with previous methods. However, given the computational complexity, the vast number of SNPs makes this algorithm hard to be applied to real GWAS data. In other words, these methods cannot meet both the accuracy and scalability requirements for genome-wide association studies. In addition to these typical approaches, some Bayesian network approaches for specific scenarios are also interesting. For example, BNOmics software can deal with heterogeneous datasets containing many data types, such as genetic data, epigenetic data, transcriptome data, epidemiological data and so on (Gogoshin et al., 2016). Zhang et al. studied whether and to what extend exploiting public GWAS statistics can be used to infer private information about general population by Bayesian network (Zhang et al., 2019).

Considering the difficulties in parameter estimation of the traditional methods and the high false positive rate of machine learning methods, Bayesian network methods seem more appropriate for detecting associated SNPs. But when learning the network structure, the stability of the constraint-based methods is affected by sample sizes, and the score-and-search methods are hard to use in high dimensional GWAS datasets. In this article, we proposed a Bayesian network method with a combination of constraint-based method and score-and-search method. First, we use the constraint-based method to get a smaller candidate set. After that, a score-and-search method is used to determine the final parent node set. Both computational feasibility and accuracy are taken into consideration in our algorithm. In simulation experiments, we compared our method with BEAM, BOOST, MOMDR, SVM, DASSO-MB and bNEAT. From the results, we can see both our method and bNEAT have good performance in terms of accuracy. However, bNEAT cannot handle the large volume of data in a real genome-wide association study, and our method is more robust than bNEAT when we randomly disturb the input SNPs sequence. In addition, we applied our algorithm to the real data from the Stanley Medical Research Institute Online Genomics Database (SMRIDB) and the UK Biobank, and finally found several SNPs associated with corresponding diseases.

## 2 Methods

### 2.1 Bayesian network

Bayesian network is a probabilistic graphical model and its structure is a directed acyclic graph (DAG), G=(V, E) , where the nodes set $V=\left(X_{1},\;X_{2},\;\ldots ,X_{n}\right)$ represents random variables, and the edges set E represents the dependence relationships between the variables. There are several properties that form the core of Bayesian network methods.

Definition 1 (***Faithfulness***) A Bayesian network G and a joint probability distribution P are faithful to each other if and only if every conditional independence relationship in G also exists in P .

Definition 2 (***Markov blanket***) The Markov blanket of variable T *,* MB(T) , is the minimal set satisfying the following condition:

∀X∈U\MB(T)\{T}, X⊥T | MB(T)

All the variables out of MB(T) are conditionally independent of T given MB(T) , and the Markov Blanket of T can shield it from the rest of the nodes in the network. That is, the Markov Blanket of a variable has all the necessary information to predict this variable. In Bayesian Network, the Markov Blanket of T contains the parent and the child nodes of T , and other parents of the children of T (Han et al., 2010).

Theorem 1 (***local Markov property***) A variable is conditionally independent of its nondescendant nodes given its parent nodeset. That is:

∀Y∈NonDes(X)\Pa(X), X⊥Y | Pa(T)

where NonDes(X) represents the nondescendant nodes set of X , and Pa(X) represents the parent nodes set of X . According to this property, the joint probability distribution of the Bayesian network can be defined as:

$$P\left(X_{1},\ldots ,X_{n}\right)=\overset{n}{\underset{i=1}{\prod }}P\left(X_{i}|Pa\left(X_{i}\right)\right)$$

where $X_{i}$ is the node in the network and $Pa\left(X_{i}\right)$ means the parents set of $X_{i}$ .

Theorem 2 Based on the assumption of faithfulness, X and Y are adjacent if and only if there is not a set Z that X ⊥Y | Z and X, Y∉Z .

Theorem 2 is an important foundation of our algorithm, which guarantees the nodes filtered out in the first stage are not adjacent to the target node, i.e., are not parents or children of the disease node.

### 2.2 Structure learning methods

Another question is how to learn Bayesian network structure. Common approaches can be divided into two types: constraint-based methods and score-and-search methods. Generally, constraint-based methods utilize the dependence and independence relationships between variables to infer the network skeleton and then determine the direction of the edge using other Bayesian network properties. Score-and-search methods transform structure learning problems into model selection problems by treating Bayesian networks as probability models. They typically consist of a score function for evaluating the fitting effect and a search algorithm. The likelihood function can be used as the score function to reflect the fitness between the model and data. However, this may lead to over-fitting due to the lack of penalty term considering the model complexity. To avoid this, we think about some other common score functions.

Comparatively speaking, score-and-search methods can obtain more accurate results than constraint-based methods. Constraint-based methods are sensitive to the error of conditional independent tests, making their results unstable, especially when the sample sizes are small. However, given a score function, finding out the correct network structure is a tough problem. An inappropriate search program can easily lead to a local optimum or an NP-hard problem. Under this circumstance, it's a natural extension to combine these two types of methods in order to improve the performance of algorithms. This is not a fresh idea in the field of network structure learning. Many hybrid methods have been developed up to now and have been shown to perform well (Acid and de Campos, 2001; Tsamardinos et al., 2006). But this idea doesn’t often occur in GWAS. When identifying associated SNPs, combining the Bayesian network method with other art-of-state algorithms seems more popular. In this paper, we proposed a two-stage method to accomplish the combination of two methods. Looser criteria provided by the constraint-based method determine a candidate nodes set. After that a score-and-search procedure is used to find out genuinely associated nodes.

### 2.3 Algorithm

In order to balance the accuracy and feasibility, our algorithm is a score-based two-stage Bayesian network (STS-BN) method, where the idea of the constraint-based method and the score-and-search method are both used. The pseudo-code is given in Table 1. We input the disease node *D* and the set *U* containing all SNP nodes and then by calculating, we can gain a disease-associated SNPs set *V*, i.e., the parent nodes set of *D*. The whole algorithm can be divided into two parts. The first phase reduces the dimension of SNPs through the idea of constraint-based methods, filtering out some nodes which are neither parents nor children of disease node *D* via Theorem 2. In this stage, we use G-test to verify the independence and conditional independence of two variables. Compared to the chi-square test, G-values are additive and can be used for more elaborate statistical designs (McDonald, 2014). The general formula for G is:

$$G=2\underset{i}{\sum }O_{i}\ln\frac{O_{i}}{E_{i}}$$

**TABLE 1:** STS-BN algorithm.

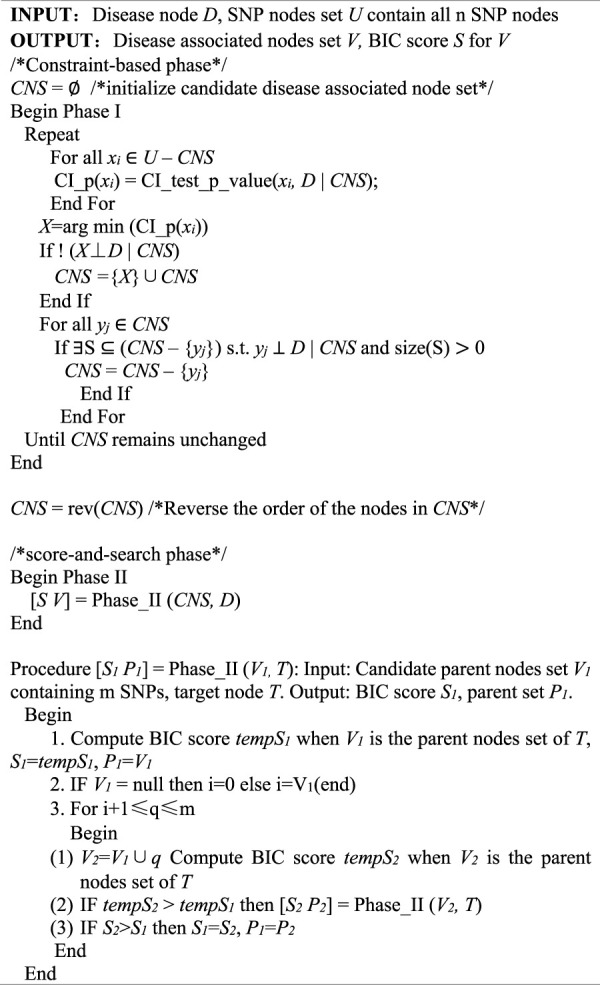

More specifically, the G-test of independence between two categorical variables A and B can be defined as

$$G=2\sum_{a,b}N_{ab}\ln\frac{N_{ab}}{E_{ab}},$$

where $E_{ab}=\frac{N_{∙b}N_{a∙}}{N_{∙∙}}$ .

The degrees of freedom for the G-test between A and B can be calculated by:

df=(Cat(A)−1)×(Cat(B)−1)

When considering conditional independence, the G-test of conditional independence between two variables A and B conditioning on a variable set C can be written as

$$G=2\sum_{a,b,c}N_{abc}\ln\frac{N_{abc}}{E_{abc}},$$

where $E_{abc}=\frac{N_{∙bc}N_{a∙c}}{N_{∙∙}c}$ .

The degrees of freedom will be:

$$df=\left(Cat\left(A\right)-1\right)\times \left(Cat\left(B\right)-1\right)\times \underset{i}{\prod }{Cat\mathrm{(C}}_{i})$$

Cat(X) represents the number of categories of variable X. The numbers of empty cells in the contingency table are reduced when calculating the degrees of freedom.

In the second phase, a score-and-search process is utilized to analyze the candidate nodes set from phase I and finally select the parents set of the disease node. We consider the Bayesian information criterion (BIC) as our score function because it's a score equivalent, decomposable, and consistent scoring criterion (Nandy et al., 2018). To cater for the circumstance that the sample sizes of GWAS data are usually not big enough on account of high research cost, we adjust the coefficient of the penalty term (0.17 here) at the suggestion of Han and Chen (2011). To save time, we embed the greedy search in phase II, which might make the algorithm sensitive to different orders of input nodes. But in fact, the first phase of our algorithm can provide a proper order while decreasing the dimension, which greatly improves the stability of the whole algorithm.

## 3 Results

### 3.1 Simulation study

#### 3.1.1 Materials

We compare the performance of our method and several other approaches using the simulated data sets generated from three common two-loci disease models (Marchini et al., 2005; Li and Chen, 2008), whose disease odds for every genotype are displayed in Table 2. In Model 1, two loci have an independent multiplicative genotype effect. In Model 2, there is also a multiplicative effect but it only appears when both two loci have the disease-associated allele. Model 3 is a typical threshold model where genotype effects appear equally as long as both two loci have the disease-associated allele.

**TABLE 2:** Two-loci disease models.

Model 1	bb	Bb	BB
Aa	α	α(1+θ)	${\alpha \left(1+\theta \right)}^{2}$
Aa	α(1+θ)	${\alpha \left(1+\theta \right)}^{2}$	${\alpha \left(1+\theta \right)}^{3}$
AA	${\alpha \left(1+\theta \right)}^{2}$	${\alpha \left(1+\theta \right)}^{3}$	${\alpha \left(1+\theta \right)}^{4}$

**Model 2**	**bb**	**Bb**	**BB**
aa	α	α	α
Aa	α	α(1+θ)	${\alpha \left(1+\theta \right)}^{2}$
AA	α	${\alpha \left(1+\theta \right)}^{2}$	${\alpha \left(1+\theta \right)}^{4}$

**Model 3**	**bb**	**Bb**	**BB**
aa	α	α	α
Aa	α	α(1+θ)	α(1+θ)
AA	α	α(1+θ)	α(1+θ)

We use α and θ to represent the baseline effect and genotype effect, respectively. For convenience sake, we introduce some parameters to reflect the data set characteristics: a marginal parameter, λ , a disease prevalence, p , the minor allele frequency (MAF), and the linkage disequilibrium, LD (measured by the parameter $r^{2}$ ). First, we specify the minor allele frequency of disease locus, λ , p and $r^{2}$ . Under the assumption of Hardy-Weinberg law, we can deduce the value of α and θ from the expressions of λ and p :

$$\lambda =\frac{p\left(D|1_{A}\right)}{p\left(\bar{D}|1_{A}\right)}/\frac{p\left(D|0_{A}\right)}{p\left(\bar{D}|0_{A}\right)}-1,$$

$$p=\underset{g_{A},{\;g}_{B}}{\sum }p\left(D|g_{A},{\;g}_{B}\right)p\left(g_{A},{\;g}_{B}\right),$$

where D represents an individual who has the disease, $\bar{D}$ represents an individual who doesn’t have disease and $g_{A},{\;g}_{B}$ are genotypes.

We can also calculate the conditional probability of the locus having linkage disequilibrium with the disease locus given the allele of the disease locus using $r^{2}$ . In population genetics, linkage disequilibrium describes a phenomenon where there are nonrandom associations between different genetic markers in a given population. The allele frequencies are written as $\pi_{C},\;\pi_{c},\;\pi_{D},\;\pi_{d}$ , and the haplotype frequencies are written as $\pi_{CD},\;\pi_{cD},\;\pi_{Cd},\;\pi_{cd}$ . Then, the expression for $r^{2}$ can be written as:

$$r^{2}\equiv \frac{{\left(\pi_{CD}-\pi_{C}\pi_{D}\right)}^{2}}{\pi_{C}\pi_{c}\pi_{D}\pi_{d}}\;.$$

Once the parameters are prepared, we can generate the disease status in a 1:1 ratio and the genotype of the disease locus. According to the genotype of disease loci, its associated loci can also be generated.

In this study, we choose 36 sets of parameters as shown in Table 3 to guarantee the generality of the experiment.

**TABLE 3:** Parameters.

	λ	$r^{2}$	MAF
Model 1	0.3	0.5, 0.7, 0.9	0.05, 0.1, 0.2, 0.5
Model 2	0.3	0.5, 0.7, 0.9	0.05, 0.1, 0.2, 0.5
Model 3	0.6	0.5, 0.7, 0.9	0.05, 0.1, 0.2, 0.5

For each parameter setting, we generate 50 datasets, each of which contains 2000 samples and 102 makers. Two markers are directly associated with the disease, and two markers are associated with these two disease-associated markers, respectively, but not directly associated with the disease. The cases and controls are generated in a ratio of 1:1. The MAF of each non-disease marker is randomly generated from a uniform distribution.

We compare our algorithm with BEAM, BOOST, MOMDR, SVM, DASSO-MB and bNEAT. To assess the outcome, we define power as the proportion of the datasets whose disease loci are detected accurately without any false positive. The powers of different methods are calculated and compared under our parameter setup.

BEAM detects the SNPs associated with disease via the Bayesian partition model. This method labels the SNPs as the members of group 0, group 1, and group 2, which contains the SNPs that have no effect on the disease, influence the disease independently, and have a joint influence on the disease with other SNPs, respectively. And then, MCMC simulations are used to estimate the posterior probability that each SNP belongs to different groups, and the B statistics are calculated for the test of significance. The software we used here is downloaded from http://www.fas.harvard.edu/∼junliu/BEAM/.

BOOST is a two-stage search method containing a screening stage and a testing stage. In the former stage, it uses a noniterative method to approximate the likelihood ratio statistic in evaluating all pairs of SNPs and select candidate SNP pairs by a threshold. Then in the testing stage, the classical likelihood ratio test is employed to measure the interaction effects of candidate SNP pairs (Wan et al., 2010). The software can be found at http://bioinformatics.ust.hk/BOOST.html.

MOMDR is a MDR-based method with a multiobjective function. This method considers the incorporated measures including correct classification and likelihood rates to detect epistatic interactions (Yang et al., 2018). The software can be found at https://goo.gl/M8dpDg.

As for support vector machines, we mimic the process in Jiang et al. (2009) instead of using the methods provided by Chen et al. (2008) for time-saving. Firstly, we rank the SNPs according to the mutual information between SNPs and disease status. Then, we select a candidate SNPs subset via a sliding window sequential forward feature selection algorithm where the accuracy rate for classification of SVM estimated by 10-fold cross-validation is used as criteria. Finally, $\chi^{2}$ tests with a Bonferroni correction are conducted to obtain the causal SNPs.

DASSO-MB is a constraint-based Bayesian network approach that uses conditional independence tests to detect the Markov blanket of the disease. The algorithm is given by Han et al. (2010) and we implement it in an R program.

bNEAT is another Bayesian network method that is designed on the basis of the score-and-search approach and is more suitable for small sample data. Although it outperforms DASSO-MB (Han and Chen, 2011), this algorithm is developed based on a greedy search program and is sensitive to improper input orders. And unfortunately, although designers try to reduce computational complexity consciously, this method still has difficulty in applying to the GWAS data directly. Its pseudo-code is shown in Han and Chen (2011) and we implement it in R, too.

#### 3.1.2 Results

**FIGURE 1:**
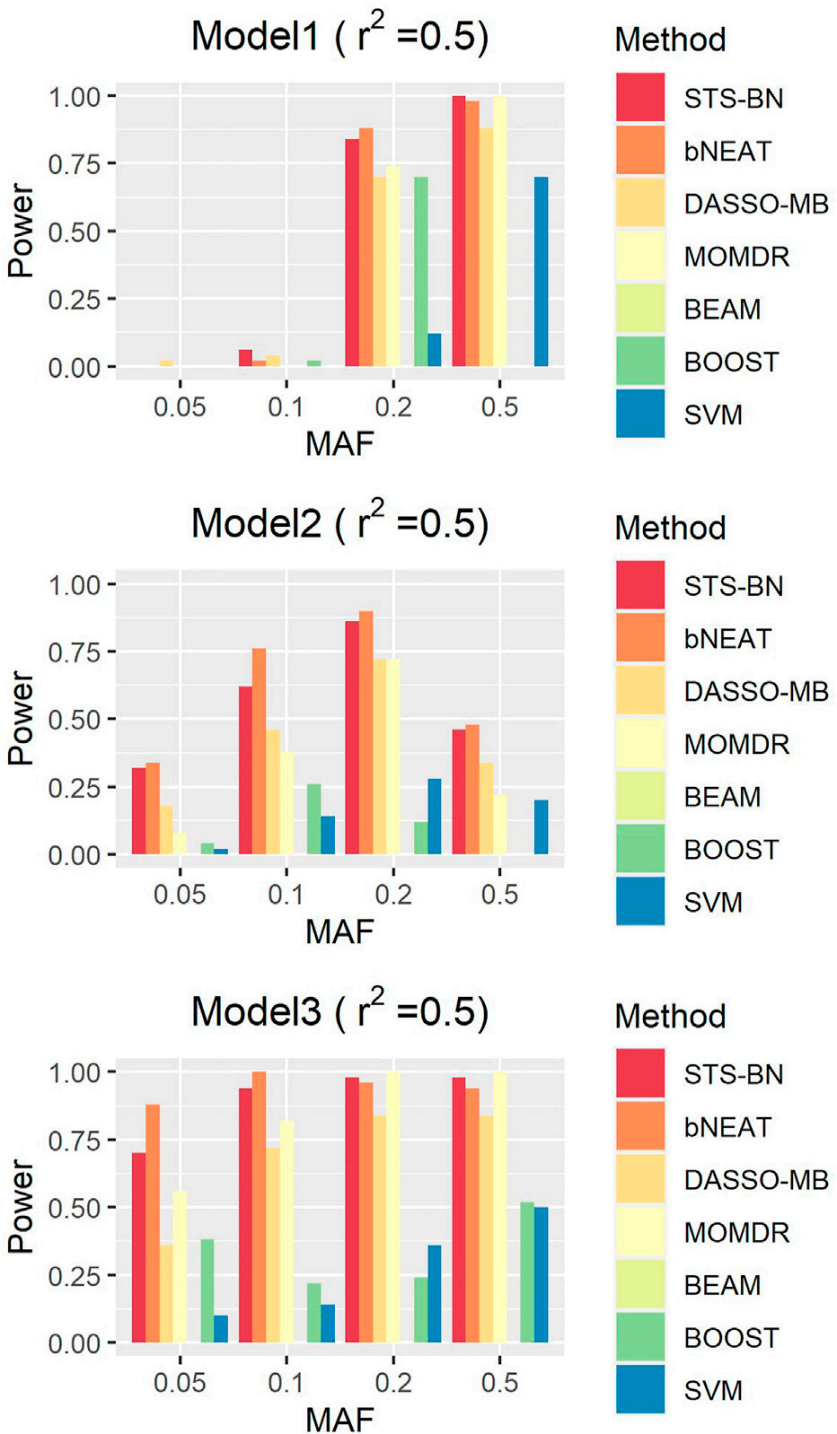
Performance comparison. Powers of STS-BN, bNEAT, DASSO-MB, MOMDR, BEAM, BOOST, SVM. ( $r^{2}$ = 0.5).

**FIGURE 2:**
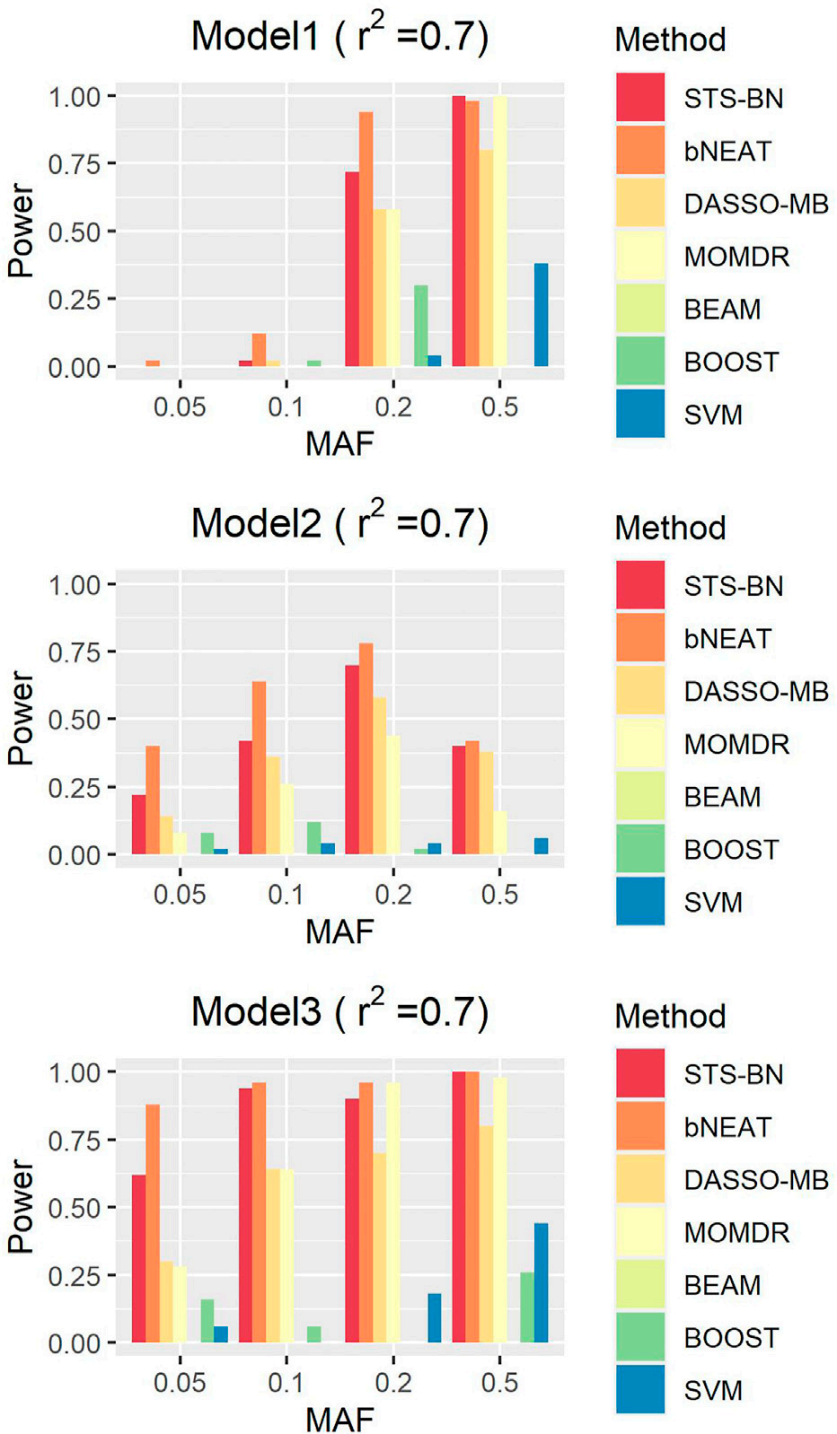
Performance comparison. Powers of STS-BN, bNEAT, DASSO-MB, MOMDR, BEAM, BOOST, SVM. ( $r^{2}$ = 0.7).

**FIGURE 3:**
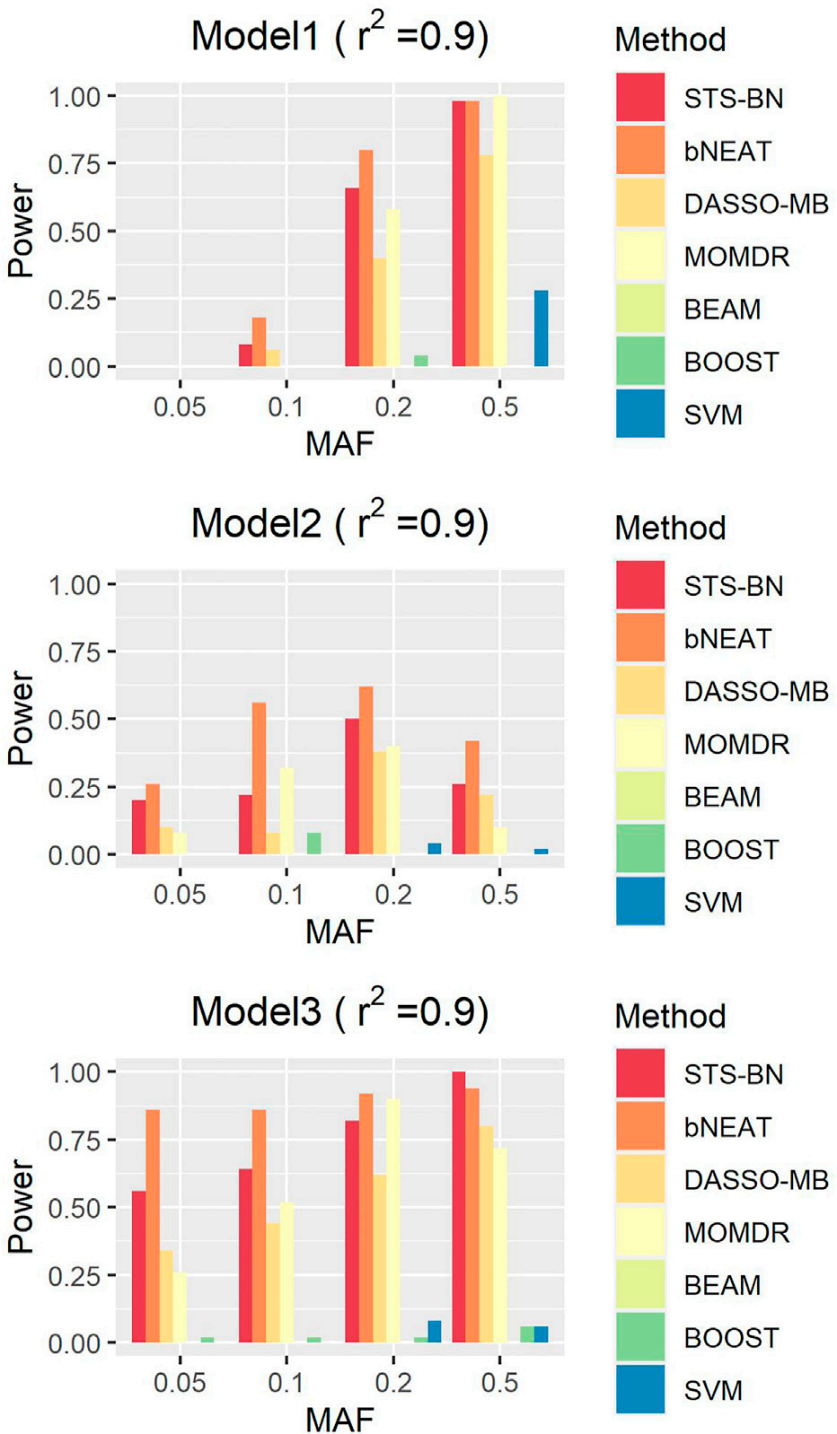
Performance comparison. Powers of STS-BN, bNEAT, DASSO-MB, MOMDR,BEAM, BOOST, SVM. ( $r^{2}$ = 0.9).

In order to quantify the performance of different methods, we define the criteria (here we name it power) as the ratio of the number of simulated datasets in which only the correct markers are detected without any false positive and the total amount of datasets under the same parameter configuration. In Figures 1–3, we use histograms to describe the simulation results. In most cases, the power of our method is closed to bNEAT, and is higher than the other methods. One of the reasons why SVM doesn’t work well might be that using prediction accuracy as the inclusive criteria of associated markers can introduce a lot of false positives. Also as listed in Table 4, in most cases, the change in power of our method is relatively small when two additional false positives are allowed to appear in the results. This suggests that our method can reduce the occurrence of false positives to some extent.

**TABLE 4:** Performance Comparison. Powers of simulations are given by this table. The percentage of the simulated datasets in which the correct markers are detected and at most two false positives are included is shown in the parentheses.

Model 1 ( $r^{2}$ = 0.5)				
MAF	0.05	0.1	0.2	0.5
STS-BN	0(0)	0.06(0.06)	0.84(0.86)	1(1)
bNEAT	0(0)	0.02(0.02)	0.88(0.9)	0.98(1)
DASSO-MB	0.02(0.08)	0.04(0.08)	0.7(0.86)	0.88(1)
MOMDR	0(0)	0(0)	0.74(0.84)	1(1)
BEAM	0(0)	0(0)	0(0)	0(0)
BOOST	0(0)	0.02(0.02)	0.7(0.98)	0(0.98)
SVM	0(0)	0(0)	0.12(0.46)	0.7(0.96)

**Model 2 (** $r^{2}$ ** = 0.5)**				
**MAF**	**0.05**	**0.1**	**0.2**	**0.5**
STS-BN	0.32(0.32)	0.62(0.62)	0.86(0.9)	0.46(0.46)
bNEAT	0.34(0.34)	0.76(0.76)	0.9(0.94)	0.48(0.48)
DASSO-MB	0.18(0.42)	0.46(0.64)	0.72(0.92)	0.34(0.46)
MOMDR	0.08(0.1)	0.38(0.46)	0.72(0.74)	0.22(0.24)
BEAM	0(0)	0(0)	0(0)	0(0)
BOOST	0.04(0.04)	0.26(0.34)	0.12(0.18)	0(0)
SVM	0.02(0.02)	0.14(0.2)	0.28(0.58)	0.2(0.44)

**Model 3 (** $r^{2}$ ** = 0.5)**				
**MAF**	**0.05**	**0.1**	**0.2**	**0.5**
STS-BN	0.7(0.74)	0.94(0.94)	0.98(1)	0.98(1)
bNEAT	0.88(0.88)	1(1)	0.96(1)	0.94(1)
DASSO-MB	0.36(0.82)	0.72(0.96)	0.84(1)	0.84(1)
MOMDR	0.56(0.6)	0.82(0.9)	1(1)	1(1)
BEAM	0(0)	0(0)	0(0)	0(0)
BOOST	0.38(0.74)	0.22(0.96)	0.24(1)	0.52(0.74)
SVM	0.1(0.2)	0.14(0.78)	0.36(0.82)	0.5(0.88)

**Model 1 (** $r^{2}$ ** = 0.7)**				
**MAF**	**0.05**	**0.1**	**0.2**	**0.5**
STS-BN	0(0)	0.02(0.02)	0.72(0.74)	1(1)
bNEAT	0.02(0.02)	0.12(0.12)	0.94(0.96)	0.98(1)
DASSO-MB	0(0.02)	0.02(0.02)	0.58(0.74)	0.8(1)
MOMDR	0(0)	0(0)	0.58(0.74)	1(1)
BEAM	0(0)	0(0)	0(0)	0(0)
BOOST	0(0)	0.02(0.02)	0.3(0.84)	0(0.98)
SVM	0(0)	0(0)	0.04(0.36)	0.38(0.86)

**Model 2 (** $r^{2}$ ** = 0.7)**				
**MAF**	**0.05**	**0.1**	**0.2**	**0.5**
STS-BN	0.22(0.22)	0.42(0.42)	0.7(0.7)	0.4(0.4)
bNEAT	0.4(0.4)	0.64(0.64)	0.78(0.82)	0.42(0.48)
DASSO-MB	0.14(0.44)	0.36(0.48)	0.58(0.7)	0.38(0.44)
MOMDR	0.08(0.1)	0.26(0.34)	0.44(0.54)	0.16(0.22)
BEAM	0(0)	0(0)	0(0)	0(0)
BOOST	0.08(0.18)	0.12(0.22)	0.02(0.14)	0(0)
SVM	0.02(0.02)	0.04(0.18)	0.04(0.5)	0.06(0.26)

**Model 3 (** $r^{2}$ ** = 0.7)**				
**MAF**	**0.05**	**0.1**	**0.2**	**0.5**
STS-BN	0.62(0.62)	0.94(0.94)	0.9(0.94)	1(1)
bNEAT	0.88(0.88)	0.96(0.96)	0.96(1)	1(1)
DASSO-MB	0.3(0.78)	0.64(0.96)	0.7(0.96)	0.8(1)
MOMDR	0.28(0.34)	0.64(0.76)	0.96(0.98)	0.98(0.98)
BEAM	0(0)	0(0)	0(0)	0(0)
BOOST	0.16(0.7)	0.06(0.94)	0(1)	0.26(0.8)
SVM	0.06(0.34)	0(0.78)	0.18(0.78)	0.44(0.88)

**Model 1 (** $r^{2}$ ** = 0.9)**				
**MAF**	**0.05**	**0.1**	**0.2**	**0.5**
STS-BN	0(0)	0.08(0.08)	0.66(0.68)	0.98(1)
bNEAT	0(0)	0.18(0.18)	0.8(0.84)	0.98(1)
DASSO-MB	0(0)	0.06(0.1)	0.4(0.68)	0.78(1)
MOMDR	0(0)	0(0)	0.58(0.7)	1(1)
BEAM	0(0)	0(0)	0(0)	0(0)
BOOST	0(0)	0(0.08)	0.04(0.94)	0(0.98)
SVM	0(0)	0(0)	0(0.32)	0.28(0.78)

**Model 2 (** $r^{2}$ ** = 0.9)**				
**MAF**	**0.05**	**0.1**	**0.2**	**0.5**
STS-BN	0.2(0.2)	0.22(0.22)	0.5(0.52)	0.26(0.26)
bNEAT	0.26(0.26)	0.56(0.56)	0.62(0.64)	0.42(0.44)
DASSO-MB	0.1(0.26)	0.08(0.28)	0.38(0.54)	0.22(0.36)
MOMDR	0.08(0.08)	0.32(0.36)	0.4(0.44)	0.1(0.12)
BEAM	0(0)	0(0)	0(0)	0(0)
BOOST	0(0.1)	0.08(0.32)	0(0.3)	0(0)
SVM	0(0.02)	0(0.08)	0.04	0.02

**Model 3 (** $r^{2}$ ** = 0.9)**				
**MAF**	**0.05**	**0.1**	**0.2**	**0.5**
STS-BN	0.56(0.56)	0.64(0.64)	0.82(0.84)	1(1)
bNEAT	0.86(0.86)	0.86(0.86)	0.92(0.96)	0.94(0.9)
DASSO-MB	0.34(0.58)	0.44(0.64)	0.62(0.84)	0.8(1)
MOMDR	0.26(0.32)	0.52(0.68)	0.9(0.94)	0.72(0.76)
BEAM	0(0)	0(0)	0(0)	0(0)
BOOST	0.02(0.84)	0.02(0.98)	0.02(0.94)	0.06(0.76)
SVM	0(0.38)	0(0.6)	0.08(0.78)	0.06(0.8)

**FIGURE 4:**
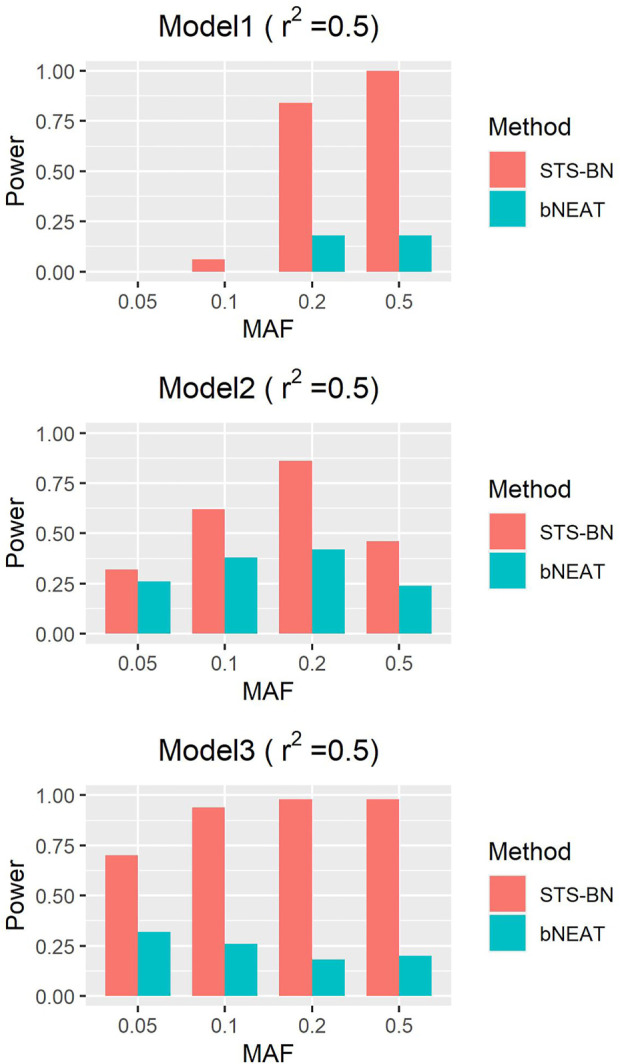
Performance comparison of STS-BN and bNEAT. The input orders of markers are randomly disturbed and $r^{2}$ = 0.5.

**FIGURE 5:**
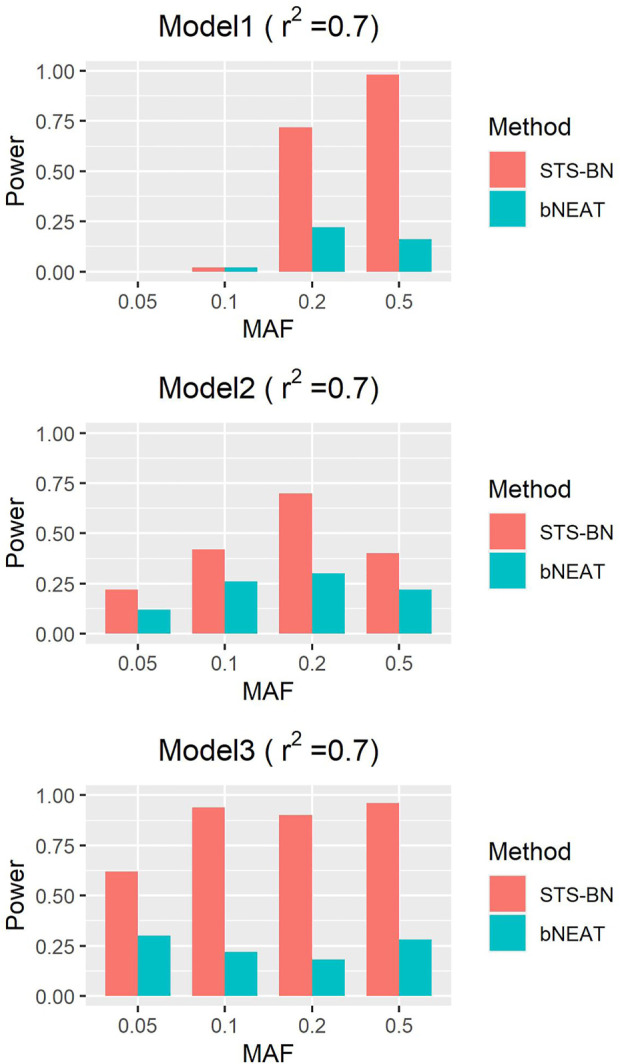
Performance comparison of STS-BN and bNEAT. The input orders of markers are randomly disturbed and $r^{2}$ = 0.7.

**FIGURE 6:**
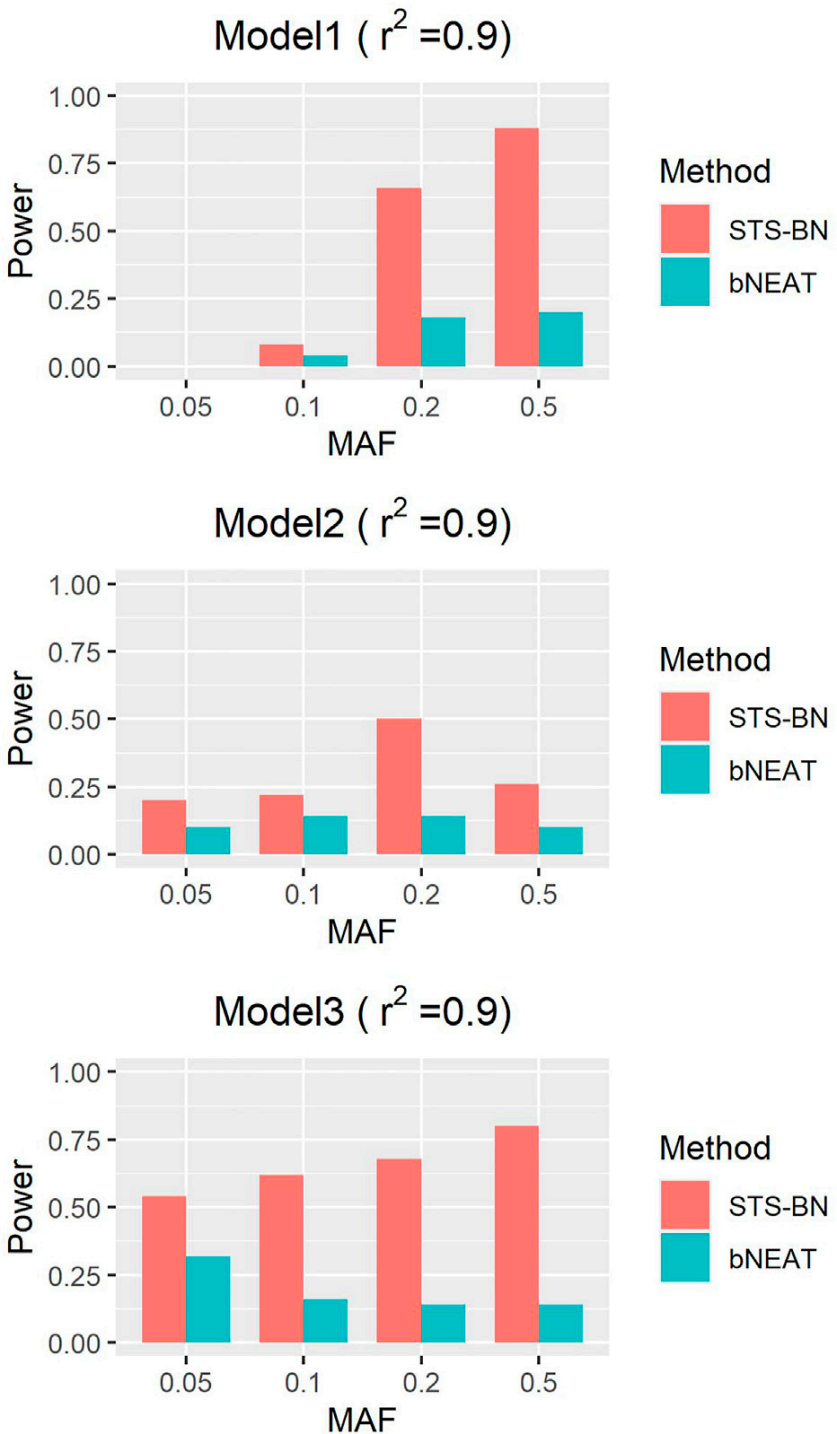
Performance comparison of STS-BN and bNEAT. The input orders of markers are randomly disturbed and $r^{2}$ = 0.9.

As mentioned above, though bNEAT usually performs well in some simulation experiments, it can hardly get good results when the input sequence is inappropriate. So, we randomly disturb the order of the makers in each dataset and reproduce the experiment. From the results in Figures 4–6, we can see that our algorithm far outperforms bNEAT. Under most configurations, the results of our method are the same as the former, while the accuracy of bNEAT is significantly reduced. This suggests that our method possesses higher stability than bNEAT.

### 3.2 Application to real datasets

#### 3.2.1 UK biobank lung cancer data

We apply our algorithm to the real data set to evaluate its adaptability to the real-world situation. The dataset we used is a lung cancer dataset extracted from the UK Biobank. The UK Biobank is a large-scale biomedical database and research resource containing in-depth genetic and health information from approximately 500,000 individuals from across the United Kingdom, aged between 40 and 69 at recruitment (Bycroft et al., 2018). Here we selected the patients with malignant neoplasm of bronchus and lung based on the ICD-10 code for the type of cancer. The controls were selected from the population, where we excluded the patients with malignant neoplasm of bronchus and lung or lung cancer based on ICD-10, ICD-9 or self-reported code, by R package “MatchIt” (Ho et al., 2011) according to the sex and age of the participants in a 1:1 ratio. Then, these people’s imputed genetic variation data on autosomes were extracted. Data preprocessing was completed by PLINK 2.0 (Chang, 2022). In this step, the variants with minor allele frequency ≤0.05 , missing call rate >0.05 , or the Hardy-Weinberg equilibrium exact test *p*-value < 1e-50 were excluded. Variants with more than 2 alleles were also filtered out. After these, we got a data set with 5472 samples (2736 cases and 2736 controls) and 5,637,802 SNPs.

Using STS-BN to analyze the dataset, rs6534554 and rs10229375 were detected. The importance of the latter has been more evident in previous researches. According to dbSNP (Sherry et al., 2001), rs10229375 is the intro variant of *STX1A* and the 2 kb upstream variant of *MIR4284*. *STX1A* encodes a member of the syntaxin superfamily. This protein has been shown to be associated with different types of lung cancer (Graff et al., 2001; Zombori et al., 2021). In another study, *STX1A* was used to classify NSCLC (non-small cell lung cancer) patients into different prognostic groups (Lau et al., 2007). In addition, a recent study suggested that the up-regulation of *MIR4284*, i.e. microRNA 4284, was shown in NSCLC tissues and cell lines compared to the corresponding normal controls, and decreased expression of *MIR4284* could inhibit tumor cell proliferation, migration and invasion (Tian et al., 2021).

#### 3.2.2 SMRIDB data

Another experiment is conducted with a dataset downloaded from The Stanley Medical Research Institute Online Genomics Database (Higgs et al., 2006), which can be found at https://www.stanleygenomics.org/. This database collected the information of patients suffering from mental diseases such as schizophrenia and bipolar disorder. Schizophrenia is a devastating and debilitating form of chronic psychiatric disorder, which is expressed as a combination of psychotic symptoms and motivational and cognitive dysfunctions. As a cognitive and behavioural disorder, schizophrenia is ultimately about how the brain processes information. Besides, this disease involves subtle pathological changes in specific neural cell populations and cell-cell communication (Kahn et al., 2015).

To identify associated SNPs, we chose the dataset whose study ID is 20, and there are a total of 153 samples and 500,568 SNPs. After removing the missing data, we selected a control group of 48 samples and a case group including 44 patients with schizophrenia. Finally, we get a dataset containing 92 samples and 330,673 SNPs. Analyzing the dataset with STS-BN, we found three associated SNPs, rs11723575, rs1120408, and rs6062361. According to dbSNP (Sherry et al., 2001), rs1120408 and rs6062361 are intron variants of the diacylglycerol kinase beta (*DGKB*) gene and protein-L-isoaspartate (D-aspartate) O-methyltransferase domain containing 2 (*PCMTD2*) gene, respectively. *DGKB* gene codes a kind of protein that can phosphorylate diacylglycerol to phosphatidic acid, thus removing diacylglycerol. Phosphatidic acid functions both in signaling and phospholipid synthesis. *DGKB* is mainly expressed in the brain, especially the amygdala, caudate nucleus, and hippocampus within the adult brain (Caricasole et al., 2002). The expression of the *PCMTD2* gene can also be found in the brain. This gene plays an important role in myelination and regulating neural differentiation. There are some clues that suggest *PCMTD2* might be related to schizophrenia. A previous study (Zarrei et al., 2019) has shown that *PCMTD2* is one of the neurodevelopmental disorders associated genes linked across more than one disorder, including schizophrenia. A case report (Kroepfl et al., 2008) also shows that the loss of the *PCMTD2* gene seems to be responsible for severe intellectual disability. As we can see from above, both *DGKB* and *PCMTD2* are closely related to the brain and have the possibility to be associated with schizophrenia. Unfortunately, there is little information about rs11723575 at present, but we can still believe that it’s an interesting site worth exploring.

## 4 Conclusion and discussion

In the era when data acquisition is getting easier and easier, the emergence and the development of data-driven methods have become a trend. Applying data-driven methods to GWAS data is crucial for understanding and predicting some complex traits. In this paper, we design a new algorithm based on Bayesian network to detect causal SNPs of complex diseases, which shows good stability and accuracy in simulation experiments. Furthermore, we have applied this algorithm to two real-world datasets and have gotten reasonable results within an acceptable time. That is, our method has the capacity to handle real GWAS data containing hundreds of thousands of SNPs.

Although the behavior of our algorithm is satisfactory, there are a few points worth discussing. For example, G-test may not be the best criteria for constraint-based structure learning methods which is used in the first phase of our algorithm. And the strategy of inclusion one by one makes it difficult to detect interactions without at least one single locus having an independent main effect. Besides, although the first phase can provide a proper input sequence for the second phase, the essence of the search program used in phase II is still a greedy search, which means the program is possible to trap in a local optimum. We will consider other measures of conditional independence in future studies. More advanced search algorithms both in Phase I and Phase II will also be adopted. Another issue is that only a few SNPs have been found in real data experiments. We speculate that this is due to our algorithm’s tendency to reduce false positives, which may result in an increase in false negatives. The lack of complexity of data preprocessing and disease classification may also have influenced the results to some extent. In addition, we can expand our study to the circumstance where gene-gene interactions and gene-environment interactions are considered at the same time so that we can have a more objective understanding of the complex disease.

## Data Availability

The code for our algorithm and all simulated data are available from https://github.com/YanranM/STS-BN. The SMRIDB dataset is download from the Stanley Medical Research Institute Online Genomics Database, which can be accessed at https://www.stanleygenomics.org. The UK Biobank dataset can be obtained from https://www.ukbiobank.ac.uk/ and our research is conducted with approved access to UK Biobank data under application ID 47192 and run ID 40687.
